# Transgenerational Effects of Maternal Water Condition on the Growth, C:N Stoichiometry and Seed Characteristics of the Desert Annual *Atriplex aucheri*

**DOI:** 10.3390/plants10112362

**Published:** 2021-11-02

**Authors:** Li Jiang, Zhibin Wen, Yunling Zhang, Zhenyong Zhao, Mohsin Tanveer, Changyan Tian, Lei Wang

**Affiliations:** 1 State Key Laboratory of Desert and Oasis Ecology, Xinjiang Institute of Ecology and Geography, Chinese Academy of Sciences, 818 South Beijing Road, Urumqi 830011, China; jiangli@ms.xjb.ac.cn (L.J.); zhibinwen@ms.xjb.ac.cn (Z.W.); zhaozhy@ms.xjb.ac.cn (Z.Z.); tianchy@ms.xjb.ac.cn (C.T.); 2 University of Chinese Academy of Sciences, Beijing 100049, China; 3 General Grassland Station of Xinjiang, Urumqi 830049, China; yunlingzhang2021@163.com; 4 Tasmanian Institute of Agriculture, University of Tasmania, Hobart, TAS 7001, Australia; mohsin.tanveer@utas.edu.au

**Keywords:** *Atriplex aucheri*, seed characteristic, stoichiometry, transgenerational effect, water

## Abstract

Water conditions directly affect plant growth and thus modify reproduction allocation. However, little is known about the transgenerational effects of water conditions on xerophytes. The desert annual *Atriplex aucheri* produces three types of seeds (A: dormant, ebracteate black seeds; B: dormant, bracteolate black seeds; C: non-dormant, bracteolate brown seeds) on a single plant. The aim of this study was to investigate the effects of low/high water treatment (thereafter progeny water treatment) on aboveground biomass, C:N stoichiometry, and offspring seed characteristics of *A. aucheri* grown from brown seeds whose mother plants were under low/high water treatment (thereafter maternal water treatment). Progeny water only affected shoot dry weight and seed allocation of type A. Under low progeny water treatment, plants from parents with low maternal water treatment had the lowest biomass. Maternal water did not significantly influence the C and N content, however high maternal water increased the C:N ratio. Maternal water treatment did not significantly affect seed number. However, plants under low maternal and progeny water treatments had the lowest weight for type B seeds. When progeny plants were under low water treatment, seed allocation of type A, type B, and total seed allocation of plants under high maternal water were significantly lower than those of plants under low maternal water. These results indicate that water conditions during the maternal generation can dramatically contribute to progeny seed variation, but the transgenerational effects depend on the water conditions of progeny plants.

## 1. Introduction

Transgenerational effects occur when biotic or abiotic environmental factors act on maternal plants and thereby influence the phenotype of progeny plants. Transmitted factors can be divided into abiotic factors and biotic factors. Abiotic factors include light, nutrient, salinity, water condition, etc. [1,2,3,4,5]. For example, when parental ramets of *Centella asiatica* were subjected to low light treatment, biomass accumulation and total stolon length of offspring ramets under maternal light conditions were significantly greater than those of offspring ramets under non-maternal light conditions [3]. Biotic factors that have transgenerational effects include competition, herbivory, symbiotic microorganisms, etc. [4,6,7,8]. For example, when growing in competition, the progeny of parental competition plants of *Polygonum persicaria* produce great shoot mass and allocate more biomass to the stem than the progeny of parents grown without competition [9]. Most prior studies of the transgenerational effects have tested the effects on progeny growth and seed characteristics of plants that produced only one seed type [10,11,12,13]. Less is known about the C:N stoichiometry and plants producing heteromorphic seeds [14]. Some evidence indicates that these transgenerational effects might be caused by molecular epigenetic mechanisms [15].

Water stress is one of the most important abiotic factors affecting plant growth and seed yield in arid regions [16,17,18]. To cope with this stress, plants respond with ecological, physiological, and biochemical changes that affect plant growth and reproduction. Some responses to water stress may be transmitted to subsequent generations. Transgenerational effects of drought on plant growth and progeny seed characteristics have been shown in several studies [2,13,19]. For example, the growth differences between barley plants with different stress histories in the maternal generation were more pronounced in progeny grown at drought condition than at optimal water availability [10]. However, the knowledge of the responses of plants with different water demand to drought stress is still incomplete. Moreover, plants respond to stress factors with ecological, physiological, and biochemical changes, and there are few studies regarding transgenerational effects of post-drought descendants.

Most plant species produce one type of seed; however, more than 200 species produce different types of seeds on a single plant [20]. These different seed types generally vary in color, dispersal, dormancy, germination, and even plant growth and reproduction [20,21]. Maternal environments can change seed ratio and allocation of heteromorphic seeds [22,23,24]. For example, plants of *Suaeda corniculata* subsp. *mongolica* allocate more biomass to seeds when they are grown in soil of the same salinity as that of their mother and this transgenerational effect on reproductive allocation is also related to maternal seed morph [25]. Thus, the production of heteromorphic seeds reflects not only current environmental conditions but also the maternal environments.

*Atriplex aucheri* in the family Chenopodiaceae (included in Amaranthaceae) is an early successional annual xerophyte growing in Gobi desert, deserts, and arid valleys [26]. Annual precipitation of these areas is 100–250 mm, but annual potential evaporation is above 2000 mm. This species is native to Xinjiang, China (Afghanistan, Kazakhstan, Turkmenistan; SW Asia, SE Europe). *Atriplex aucheri* is an optimal organism for studying transgenerational effects of water stress because it is a typical xerophyte and a heterocarpic species producing three types of fruits and seeds on a single plant [27]. Type A seeds are small and black with a 5-lobed perianth. Type B seeds are medium-sized and have a similar appearance to type A, but they are covered by extended bracteoles. Type C seeds are large and brown and covered by extended bracteoles. Type C seeds of *A. aucheri* are non-dormant and have a high germination percentage, whereas type A and B seeds have non-deep physiological dormancy and low germination percentages [27].

We hypothesized that exposure to water stress of maternal plants of *A. aucheri* makes progeny plants more resistant to future exposure by stress memory to ensure reproductive success. Specifically, we addressed the following questions: (1) Does maternal water treatment significantly change the C content, N content, and C:N ratio? (2) What is the effect of maternal water treatment on the seed production of progeny plants? (3) Do water treatments to maternal plants and progeny plants have an interaction?

## 2. Results

### 2.1. Shoot Dry Weight

**Figure 1:**
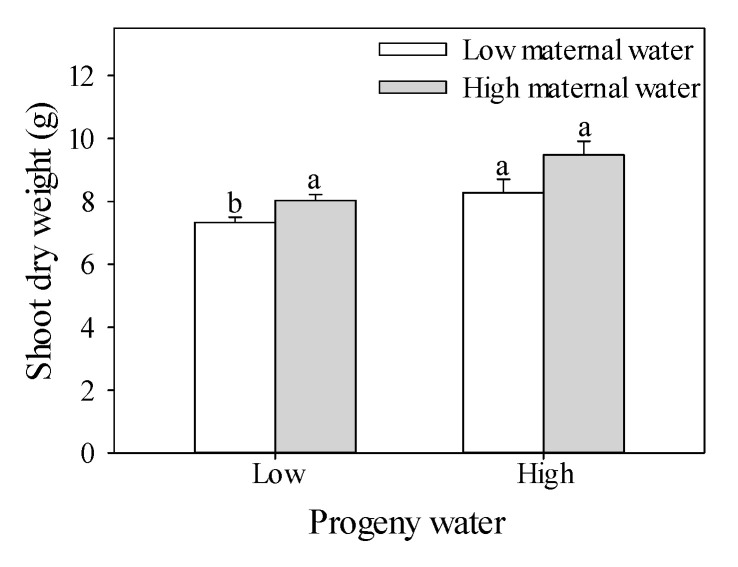
Effects of maternal and progeny water treatments on shoot dry weight per plant. Different lowercase letters represent significantly different means between plants with different maternal water treatments under the same progeny water treatment according to *t*-test (*p* < 0.05).

A two-way ANOVA showed that shoot dry weights were significantly affected by water treatment experienced by maternal plants and progeny plants (Table 1). When maternal plants were subjected to high water treatment, the shoot dry weight of progeny plants experiencing maternal water environment significantly increased relative to those of progeny plants experiencing low water treatment. Shoot dry weight of progeny plants experiencing low maternal water environment treated with low water had the lowest weight. Under low water treatment, compared with plants with maternal low water treatment, the shoot dry weight of plants with maternal high-water treatment increased 12.8%; under high water treatment, compared with plants with maternal low water treatment, the shoot dry weight of plants with high maternal water treatment increased 18.2% (Figure 1).

### 2.2. C Content, N Content and C:N Ratio

**Figure 2:**
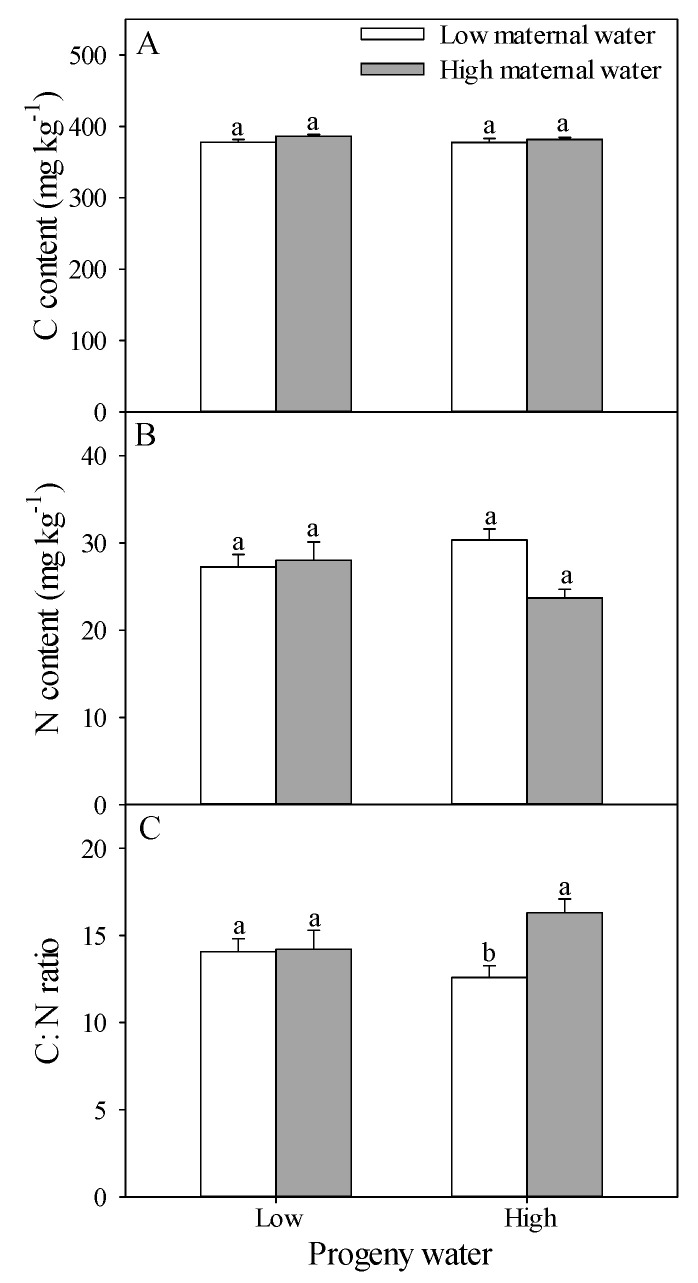
Effects of maternal and progeny water treatments on (**A**) C content, (**B**) N content, and (**C**) C:N ratio. Different lowercase letters represent significantly different means between plants with different maternal water treatments under the same progeny water treatment according to *t*-test (*p* < 0.05).

C content was not significantly affected by water treatment for maternal plants and progeny plants and their interaction (Table 1). C content ranged from 377.44 mg kg^−1^ to 386.57 mg kg^−1^ (Figure 2A).

N content was significantly affected by the interaction of water treatments for maternal plants and progeny plants, but not by the water treatment for maternal plants or water treatment for progeny plants (Table 1). The lowest value (23.68 mg kg^−1^) for N content was from the progeny plants with high water treatment grown from seeds on maternal plants with high water treatment (Figure 2B).

C:N ratio was significantly affected by the water treatment for maternal plants and the interaction between water treatments for maternal plants and for progeny plants, but not affected by water treatment for progeny plants (Table 1). The highest value (16.30) for C:N ratio was from the progeny plants with high water treatment grown from seeds on maternal plants with high water treatment (Figure 2C).

### 2.3. Seed Traits

**Figure 3:**
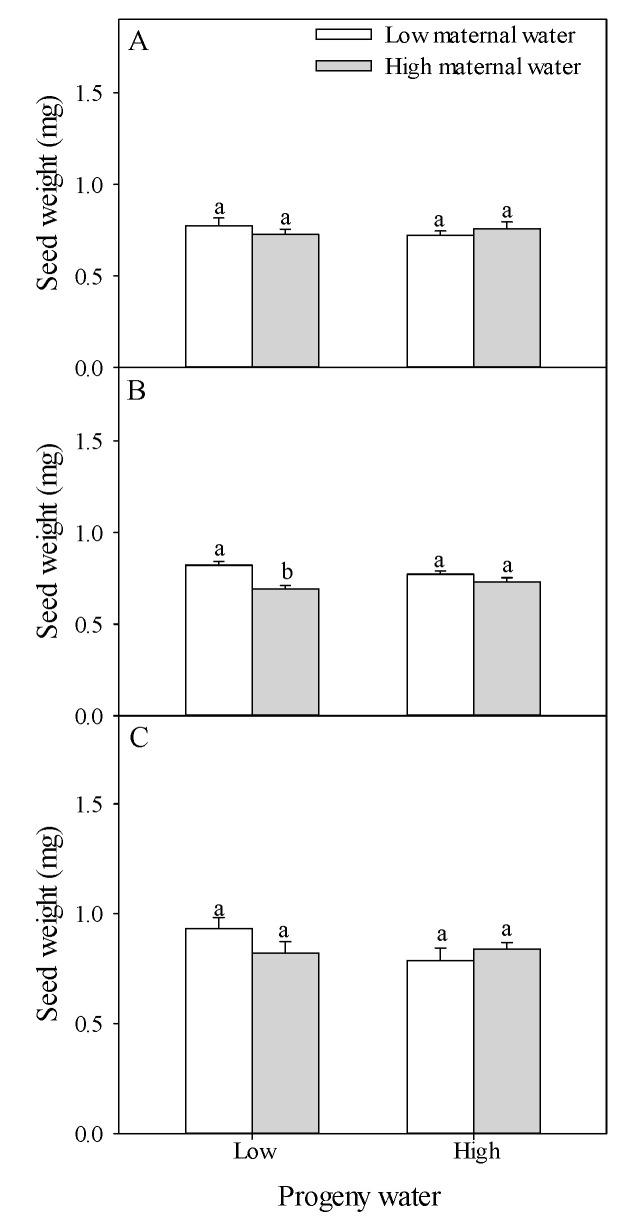
Effects of maternal and progeny water treatments on (**A**) seed weight of type A, (**B**) seed weight of type B, and (**C**) seed weight of type C. Different lowercase letters represent significantly different means between plants with different maternal water treatments under the same progeny water treatment according to *t*-test (*p* < 0.05).

**Figure 4:**
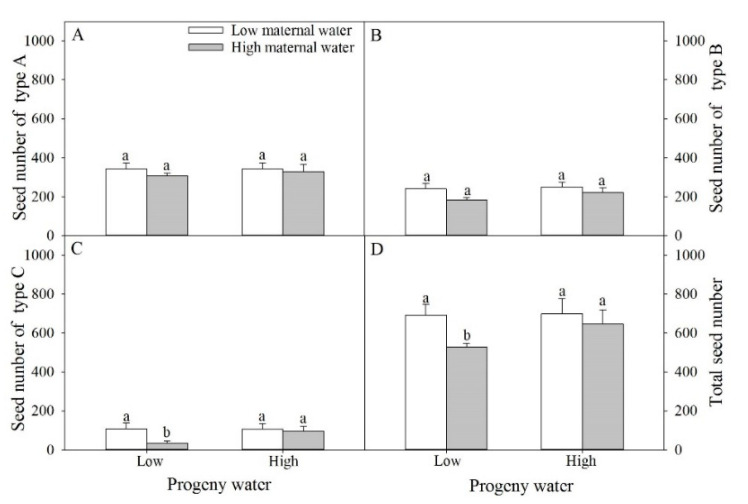
Effects of maternal and progeny water treatments on (**A**) seed number of type A, (**B**) seed number of type B, (**C**) seed number of type C, and (**D**) total seed number per plant. Different lowercase letters represent significantly different means between plants with different maternal water treatments under the same progeny water treatment according to *t*-test (*p* < 0.05).

Seed numbers of type A, type B, type C and total seed number were not significantly affected by water treatment for maternal plants and for progeny plants, and their interaction (Table 1 and Figure 3). When progeny plants were under low water treatment, compared with plants under low maternal water, seed number of type C, and total seed number of plants under high maternal water decreased 66.54% and 23.93%, respectively (Figure 4C,D).

Seed weights of type A and type C were not significantly affected by water treatment for maternal plants and for progeny plants, and their interaction, but seed weight of type B was significantly affected by water treatment for maternal plants, and their interaction (Table 1). When progeny plants were under low water treatment, compared with plants under high maternal water, seed weight of type B of plants under low maternal water increased 18.70% (Figure 3B).

**Figure 5:**
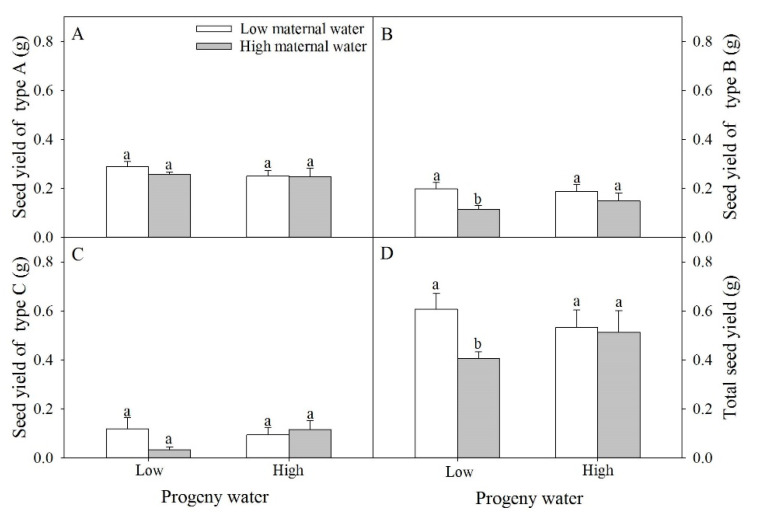
Effects of maternal and progeny water treatments on (**A**) seed yield of type A, (**B**) seed yield of type B, (**C**) seed yield of type C, and (**D**) total seed yield per plant. Different lowercase letters represent significantly different means between plants with different maternal water treatments under the same progeny water treatment according to *t*-test (*p* < 0.05).

Seed yield of type A, type C, and total seed yield were not significantly affected by water treatment for maternal plants and for progeny plants and their interaction (Table 1 and Figure 5). Seed yield of type B was significantly affected by water treatment for maternal plants (Table 1 and Figure 5B). When progeny plants were under low water treatment, seed yield of type B and total seed number of plants under high maternal water were significantly lower than that of plants under low maternal water (Figure 5B,D).

**Figure 6:**
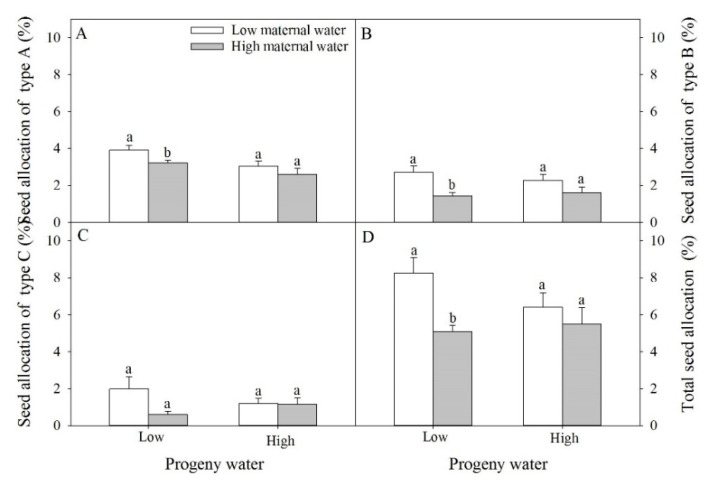
Effects of maternal and progeny water treatments on (**A**) seed allocation of type A, (**B**) seed allocation of type B, (**C**) seed allocation of type C, and (**D**) total seed allocation. Different lowercase letters represent significantly different means between plants with different maternal water treatments under the same progeny water treatment according to *t*-test (*p* < 0.05).

Seed allocation of type A was significantly affected by water for maternal plants and for progeny plants (Table 1). Seed allocations of type B and total seed allocation were significantly affected by water for maternal plants. Seed allocation of type C was not significantly affected by water for maternal plants and for progeny plants and their interaction. When progeny plants under low water treatment, seed allocation of type A, type B and total seed allocation of plants under high maternal water were significantly lower than that of plants under low maternal water (Figure 6A,B,D).

## 3. Discussion

Although seed germination ecology, plant growth, and utilization of xerophytes, for example, *A. aucheri*, have been studied extensively [28,29,30], these data are the first addressing transgenerational effects of low/high water treatment on C:N stoichiometry and progeny seed characteristics of xerophytes. In addition, our data indicated that though the maternal water treatment significantly influences shoot dry weight and C:N ratio, though the treatment does not significantly affect C and N contents. Maternal water treatment affects seed weight and seed yield of type B of progeny plants. Water treatments to maternal plants and progeny plants have an interaction on N content, C:N ratio, and seed weight of type B. The results indicated that exposure to water stress of maternal plants of *A. aucheri* makes progeny plants more resistant to future exposure to ensure reproductive success.

Transgenerational effects of drought stress have been well documented [2,10,31]. However, there are few studies about transmitted developmental effects on xerophytes. We found that maternal water conditions induced significant effects on progeny growth, C:N ratio, and seed allocation. Moreover, the drought stress imposed transgenerational effects on progeny growth and reproduction that were distinct from the mesophytes [2,12,31]. Though researches of other plant types are needed to test the transgenerational effects, such effects might be important in xerophytes, which are characterized by extreme drought conditions [32]. Our study provided new insight into transgenerational effects of water treatment by demonstrating substantial developmental effects on C:N ratio and seed allocation of different seed types.

Variation in biomass allocation to vegetative and reproductive parts is a crucial life history strategy for annuals in response to the changing environmental conditions, especially for frequently disturbed and environment extreme habitats [14,25]. Our results showed that both maternal water condition and progeny water condition could significantly influence the aboveground biomass of progeny plants of *A. aucheri*. This indicates that even for the xerophytes, a high level of maternal and progeny water is beneficial for the growth of progeny plants. However, the increase of biomass did not result in the increase of seed number and seed yield of different seed types. Contrarily, when progeny plants were cultured under low water treatment, seed yield was higher when the maternal plants were cultured under low water treatment. Considering the habitat of this xerophyte is deserts and arid valleys, the transgenerational effects of drought for progeny in similar conditions might be a kind of reproductive assurance.

Previous studies showed that plants generally have higher C:N ratio under drought stress [33]. Our results showed that C:N ratio of progeny plants were low and not significantly affected by progeny water treatment, but by the maternal water treatment. The low C:N ratio was also found in other plants grown in arid land [34]. Under high progeny water treatment, plants treated with low maternal water had lower C:N ratio than plants treated with high maternal water. The lower C:N ratio in progeny of low water-treated mothers may be due to shoots containing more metabolic, N-rich tissue. This might be an adaptation to cope with unusual high-water condition in arid land.

The seed traits of *A. aucheri* showed different responses to water treatment for maternal plants and for progeny plants. Our results indicated that water conditions had transgenerational effects on seed weight and seed yield of type B, seed allocation of type A and type B, and total seed allocation. When progeny plants were under low water treatment, seed allocation of type A, type B, and total seed allocation of plants under low maternal water were significantly higher than those of plants under high maternal water. Thus, transgenerational effects on offspring seed traits varied depending on maternal and progeny water environments. Plants generally allocate a certain proportion of resources to seeds. Thus, there is a trade-off between seed number and seed weight [35]. Our results support this optimization model as different maternal and progeny water treatments had no effect on seed number and seed weight, except the seed weight of type B. Under low progeny water treatment, plants grown from seeds with low maternal water treatment had significantly higher seed allocation, especially for type A and type B. The results indicate that under drought stress, maternal fitness of *A. aucheri* is increased by increasing offspring fitness.

## 4. Conclusions

In conclusion, this study showed that the maternal water condition can induce transgenerational effects on offspring seed allocation of seed-heteromorphic xerophyte. The allocation depends on water conditions in both maternal and offspring generations. Maternal low water conditions conferred a seed allocation advantage when offspring plants were under low water conditions. Interestingly, the transgenerational effects on offspring seed characteristics of different types were different. Importantly, for most measured indexes, there was no interaction between maternal water and progeny water treatments, suggesting both water treatments influenced the progeny in different ways. Further research should pay more attention to the transgenerational effects among multiple generations and the progeny grown from different types of seeds.

## 5. Materials and Methods

### 5.1. Seed Collection

**Figure 7:**
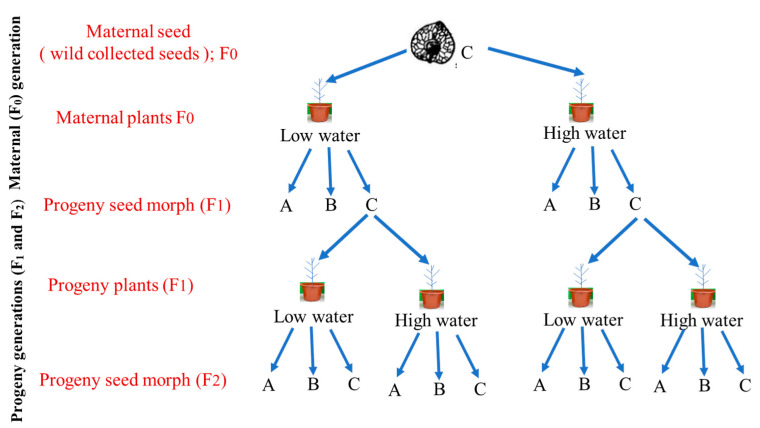
Experimental design for plants and seeds of maternal generation and progeny generations. A, B, and C indicate the seeds of type A, type B, and type C, respectively.

Seeds (F1) of *A*. *aucheri* sowed in this experiment was collected from a previous pot experiment carried out from early April 2011 to early October 2011. The experimental design constituted low and high-water treatments (Figure 7). The experimental location and methods were the same as the current pot experiment. Thus, the description for previous pot experiment was given in the methods of current experiment. After removing the bracteoles, seeds were sorted according to the water treatment and seed types, and then stored in paper bags at room temperature. Since type C seeds are non-dormant and have the highest germination percentages (usually up to 95%; [27]), they were used in the current pot experiment (Figure 1).

Seeds (F_0_) of *A. aucher* sowed in previous pot experiment were collected from a natural population growing in the hilly desert on the southern edge of Junggar Basin in Xinjiang in October 2010. Seeds were sorted according to seed types and only type C seeds were used in the previous pot experiment.

### 5.2. Pot Experiment

About 20 type C seeds (F_1_) from low and high-water treatments were sown at 2 mm soil depth into individual plastic pots (17 cm deep and 16 cm in diameter) filled with 2 L loam soil and vermiculite mixture (3:1 *v*/*v*) on 15 April 2012. For fertilization, a commonly available granular lawn fertilizer (Osmocote 301, Scotts, Marysville, OH, USA, 15N: 11P: 13K: 2 Mg) and a commercial nutrient solution (Peters1, Scotts, Marysville, OH, USA, 20N: 20P: 20K) were used. Each pot received 6 g Osmocote 301 once before sowing as basic fertilizer. The seedlings were thinned to one per pot, 20 days after sowing. To reduce variation in initial seedling size, only seedlings of a similar height for each seed type were kept. For water treatment, each pot received 100 mL water (low) and 200 mL water (high) per day. A randomized block design with ten replicates was used. Each block contained 4 pots representing a combination of two water treatments for maternal plants and two treatments for progeny plants. The experiment was terminated on 14 October 2012, and then the shoot and progeny seeds (F_2_) were harvested.

### 5.3. Shoot Dry Weight

After harvesting, shoot dry weight was recorded after oven drying at 60 °C until constant weight was obtained.

### 5.4. C Content, N Content and C:N Ratio

Before chemical analysis, the dried shoot materials were ground in a ball mill, and then passed through a 0.25 mm sieve. The ball mill was cleaned completely after each sample milling. The shoot C content and N content were determined using an elemental analyzer (FLASHEA 1112 Series CNS Analyzer, Thermo, Waltham, MA, USA).

### 5.5. Seed Traits

The number of seeds for different seed types on a single plant was counted after detached and cleaned. Seed weight of 5 randomly selected seeds for each morph from each plant were measured by using electronic balance (Precision = 0.00001 g; Sartorius, SQP). Seed yield of different seed types for each individual plant was measured by using electronic balance (Precision = 0.0001 g; Chyo Balance, JPN-200W). Seed allocation (%) = (seed yield/shoot dry weight) × 100%.

### 5.6. Statistical Analyses

Statistical analysis was performed using the SPSS software (version 16.0). All data were expressed as mean ± standard error (S.E). Two-way ANOVA was used to determine the significant effects of water treatment for progeny plants and for maternal plants, and their interaction on shoot dry weight, C content, N content, C:N ratio, seed number, seed weight, seed yield, and seed allocation.

## Figures and Tables

**Table 1:** F values from two-way ANOVA for the effects of progeny water, maternal water, and their interactions on investigated properties of *Atriplex aucheri*.

Source	Progeny Water	Maternal Water	Progeny Water × Maternal Water
Shoot dry weight (*n* = 10)	12.94 **	8.47 *	0.58
C content (*n* = 6)	0.48	2.95	0.26
N content (*n* = 6)	0.01	3.87	6.04 *
C: N ratio (*n* = 6)	0.01	5.32 *	4.54 *
Seed number of type A (*n* = 10)	0.12	0.75	0.15
Seed number of type B (*n* = 10)	0.94	3.10	0.38
Seed number of type C (*n* = 10)	1.31	2.61	1.48
Total seed number (*n* = 10)	1.06	3.22	0.87
Seed weight of type A (*n* = 50)	0.12	0.03	1.53
Seed weight of type B (*n* = 50)	0.06	17.62 ***	4.75 *
Seed weight of type C (*n* = 50)	1.73	0.37	2.85
Seed yield of type A (*n* = 10)	0.97	0.53	0.35
Seed yield of type B (*n* = 10)	0.22	5.56 *	0.72
Seed yield of type C (*n* = 10)	0.97	1.70	1.50
Total seed yield (*n* = 10)	0.60	2.70	1.82
Seed allocation of type A (*n* = 10)	7.93 **	4.49 *	0.24
Seed allocation of type B (*n* = 10)	0.19	10.28 **	0.95
Seed allocation of type C (*n* = 10)	0.15	1.39	2.73
Total seed allocation (*n* = 10)	0.92	7.23 *	2.20

Note: * *p* < 0.05, ** *p* < 0.01, *** *p* < 0.001.

## Data Availability

Data is contained within the article.
